# Modeling the Thermoelastic Bending of Ferric Oxide (Fe_2_O_3_) Nanoparticles-Enhanced RC Slabs

**DOI:** 10.3390/ma16083043

**Published:** 2023-04-12

**Authors:** Zouaoui R. Harrat, Mohammed Chatbi, Baghdad Krour, Marijana Hadzima-Nyarko, Dorin Radu, Sofiane Amziane, Mohamed Bachir Bouiadjra

**Affiliations:** 1 Laboratoire des Structures et Matériaux Avancés dans le Génie Civil et Travaux Publics, University of Djillali Liabes, Sidi Bel Abbes 22000, Algeria; 2 Institut Pascal, UMR 6602, Clermont Auvergne University, CNRS, Sigma, 63000 Clermont-Ferrand, France; 3 Department of Civil Engineering, Josip Juraj Strossmayer University of Osijek, Vladimira Preloga 3, 31000 Osijek, Croatia; 4 Faculty of civil engineering, Transilvania University of Brașov, Turnului Street No.5, 500152 Brașov, Romania; 5 Thematic Agency for Research in Science and Technology (ATRST), 16004 Algiers, Algeria

**Keywords:** nanotechnology, reinforced concrete, thermoelastic bending, mechanical bending, ferric oxide nanoparticles, homogenization, plate theory

## Abstract

Nanoparticles, by virtue of their amorphous nature and high specific surface area, exhibit ideal pozzolanic activity which leads to the formation of additional C-S-H gel by reacting with calcium hydroxide, resulting in a denser matrix. The proportions of ferric oxide (Fe_2_O_3_), silicon dioxide (SiO_2_), and aluminum oxide (Al_2_O_3_) in the clay, which interact chemically with the calcium oxide (CaO) during the clinkering reactions, influence the final properties of the cement and, therefore, of the concrete. Through the phases of this article, a refined trigonometric shear deformation theory (RTSDT), taking into account transverse shear deformation effects, is presented for the thermoelastic bending analysis of concrete slabs reinforced with ferric oxide (Fe_2_O_3_) nanoparticles. Thermoelastic properties are generated using Eshelby’s model in order to determine the equivalent Young’s modulus and thermal expansion of the nano-reinforced concrete slab. For an extended use of this study, the concrete plate is subjected to various mechanical and thermal loads. The governing equations of equilibrium are obtained using the principle of virtual work and solved using Navier’s technique for simply supported plates. Numerical results are presented considering the effect of different variations such as volume percent of Fe_2_O_3_ nanoparticles, mechanical loads, thermal loads, and geometrical parameters on the thermoelastic bending of the plate. According to the results, the transverse displacement of concrete slabs subjected to mechanical loading and containing 30% nano-Fe_2_O_3_ was almost 45% lower than that of a slab without reinforcement, while the transverse displacement under thermal loadings increased by 10%.

## 1. Introduction

Nanotechnology has become one of the most important and exciting areas of many fields such as physics, chemistry, biology, and, very recently, civil engineering. It has also attracted a great deal of attention in the field of material science and technology. To contribute in the development of concrete physical and mechanical conventional properties, researchers intent to replace long-used pozzolanic powder additives in concrete composition, such as micro silica fume (MS), blast furnace slag, and fly ash, with new materials to achieve a more efficient and durable concrete while considering the economy aspects [1,2].

Intrinsically, the specific objective of researchers lately was to find the preference for how to use nanoparticles with amorphous form to add even more capabilities to a concrete matrix. The required improvement of concrete properties reinforced with nanoparticles is mostly to be achieved due to the unique properties of those nano-sized elements, such as their high strength, elevated modulus of elasticity, high specific surface area, exorbitant electrical conductivity, enhancement of calcium silicate gel (C-S-H), and specific chemical activities (Bahari et al. [3], Bartos et al. [4], Bittnar et al. [5], Sikora et al. [6]).

On the experimental level, several studies have been carried out over the last decades to test the mechanical performance of structures enhanced by incorporating nanoparticles in concrete matrix, namely silicon dioxide SiO_2_ (Senff et al.) [7], titanium dioxide TiO_2_ (Feng et al. [8]), zinc dioxide ZnO_2_ (Nazari et al.) [9], and iron oxide Fe_2_O_3_ (Nazari et al.) nanoparticles [10]. For example, Rong et al. [11] demonstrated that adding nano-SiO_2_ to concrete matrix resulted in higher compressive and better tensile and flexural properties; due to their size in the range of 1–500 nm, they produced shorter setting time and water permeability, as well as strong resistance to chemical attacks. Because of this, they can form part of many concrete structures we see nowadays. Moreover, other considerable experimental studies have shown that the addition of silica nanoparticles SiO_2_ improves not only the mechanical characteristics but also the physio-chemical characteristics of the fresh and hardened concrete [12]. Using an experimental approach, Mondal and his colleagues [13] confirmed that silica nanoparticle reinforcement is not only better for the environment but also yields better results. It can increase the durability and performance of concrete as well as save cement and lower the project’s overall cost and environmental impact. However, the exorbitant price of nanoparticles may limit their utilization. Nazari et al. [10,14] revealed the chemical effect of incorporating oxidized nanoparticles in indoors fresh cement by increasing the degree of hydration, which leads to a greater consistency and workability of the cement paste. Ghannam et al. [15] conducted an experimental study to explore the possibility of using granite powder and iron powder to partially replace sand in concrete. The percentages of granite powder and iron powder added to replace the sand were 5%, 10%, 15%, and 20% of the sand by weight. It has been observed through experiments that the substitution of up to 20% of sand by weight with iron powder in concrete has resulted in an increase in the compressive and flexural strength of concrete [16]. Lee et al. [17] studied the effect of iron oxide additives on concrete interlocking blocs (CIBs). His study concluded a definite relationship between the flexural strength and the absorption rate of pigment-stained blocks. Kaïkea et al. [18] conducted an experimental study on six different sets of high performance fiber-reinforced concrete specimens by adding different types of mineral additives and checked their influence on the overall behavior of the material. The obtained results showed that by adding high volume content of fibers and iron oxide powder in the mix, the HPFRC material achieved very good mechanical performance.

Theoretically speaking, only a minority of research has been exposed using analytical modeling for the reinforcement of concrete structures with nanoparticles. Harrat et al. [19] conducted an analytical study of concrete beams incorporated with nano-silica particles using the Voigt model to describe the agglomeration effect of silica nanoparticles in the matrix and then studied the static behavior of nano-SiO_2_ reinforced concrete beams resting on the Winkler–Pasternak foundation. When Chatbi et al. [20] also investigated the bending behavior of SiO_2_-reinforced concrete slabs resting on Kerr’s elastic foundation, the most findings of those studies indicate that the use of an optimum amount of SiO_2_ nanoparticles on concretes increases better mechanical behavior. In addition, they concluded that the elastic foundation has a significant impact on the bending of concrete structures. Amnieh et al. [21] compared experimentally and theoretically the dynamic analysis of SiO_2_ nanoparticle mixed concrete blocks subjected to blast loading using Mindlin’s plate deformation theory (FSDT) and proved that the experimental model and the theoretical one were very close. Shokravi [22] analytically investigated the nonlinear vibration of nano-silica reinforced concrete columns based on Timoshenko’s beam (CBT) using the Mori–Tanaka homogenization model and then used the Differential Quadrature Method (DQM) to predict the frequency of the system.

Many authors have been looking for ways to promote higher-order shear deformation theories (HSDT) to satisfy equilibrium conditions at the top and bottom of the plate without using shear correction factors (Reddy [23], Touratier [24], Soldatos [25], Mantari et al. [26]) and stay to this day very efficient. However, more importantly, on the accuracy of predicting the plate response, a recent series of articles developed a new refined and robust plate theory for the mechanical behavior of a simply supported plate with only four unknown variables. Bachir Bouiadjra et al. [27], Bourada et al. [28], and Kettaf et al. [29] used the refined theory for studying the thermal buckling of functionally graded sandwich plates. Zenkour et al. [30] studied the thermoelastic bending analysis of functionally graded ceramic–metal sandwich plates, using several kinds of sandwich plates and taking into account the symmetry of the plate and the thickness of layers. Tounsi et al. [31] used a refined trigonometric shear deformation theory to analyze the thermoelastic bending of functionally graded sandwich plates. Sayyad et al. [32] have also investigated the thermoelastic bending but on laminated composite plates according to various shear deformation theories. Radwan [33] presented a hyperbolic displacement model to investigate the non-linear hygrothermal and mechanical buckling responses of FG sandwich plates resting on two-parameter elastic foundations while considering a non-linear Fourier temperature distribution as a special case. Hellal et al. [34] proposed a new simple “four-variable shear deformation” plate model to demonstrate the hygrothermal environment effects on dynamic and buckling of functionally graded material sandwich plates supported by Winkler–Pasternak elastic foundations.

In order to increase the strength and improve the mechanical properties of concrete, first the industrial iron wastes that pollute the environment were recovered and evaluated; then their chemical composition and their use in different forms in the concrete mix were analyzed.

Regarding the reinforcement of concrete by iron oxide nanoparticles, analytical studies are not found in the literature contrary to the experimental researches [35,36], and, therefore, there is a great lack in this subject despite the attractiveness of the concept of using Fe_2_O_3_ nanoparticles in enhancing concrete. For this purpose, the authors aim through this paper to present a mathematical model simulating an equivalent system. In this study, thermoelastic bending analysis is conducted to analyze the behavior of concrete slabs reinforced with ferric oxide and subjected to different varying thermal loads. A recently developed refined trigonometric shear plate theory (RTSDT) is adopted in this work to simulate plate deformations. The theory contains only four unknown functions, against five in the case of other shear deformation theories. The transverse shear stresses vary parabolically through the thickness, which satisfies the free surface conditions without shear stress. The equilibrium equations are obtained based on the principle of virtual work. Using Navier’s analytical solutions for thermoelastic bending, the effects of different parameters such as volume percent of nanoparticles, thermal loading type, and geometry parameters on the stress and displacement analysis of the plate are determined and presented.

## 2. Homogenization Model

**Figure 1:**
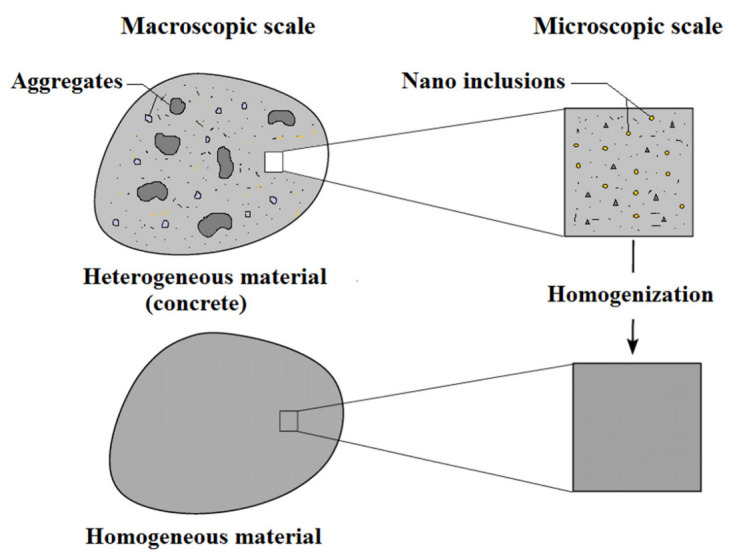
Homogenization of concrete reinforced with nanometric inclusions.

Homogenization approaches are widely used today to determine the effective properties of heterogeneous mediums. They introduce two distinct scales: the microscopic scale, where the heterogeneity of the materials is obvious, and the macroscopic scale (engineering scale), where the materials are considered homogeneous (Figure 1). Moreover, the scales must be strictly separated. This results in homogenized properties that do not particularly depend on the size of the microstructural elements. The effective properties depend only on the structural morphology and phase properties. In this paper, the analytical approach aims to determine the thermoelastic properties of reinforced concrete on the Representative Volume Element (R.V.E) scale using Eshelby’s homogenization model [37], which is limited to the average properties of composite materials with simple microstructures.

Eshelby’s homogenization model is basically the most important analytical model for predicting the properties of nano-composite reinforced matrix. Strictly speaking, it only applies to the placement of an ellipsoidal inclusion in an infinite matrix; therefore, in this study, the iron nanoparticles are considered of a spherical form. The stiffness tensor CT for the nano-composite is given by Equation (1):

(1) $$C_{T}={\left(C_{m}^{-1}-V_{r}{\left\{\left(C_{r}-C_{m}\right)\left[S-V_{r}\left(S-I\right)\right]+C_{m}\right\}}^{-1}\left(C_{r}-C_{m}\right)C_{m}^{-1}\right)}^{-1}$$

in which *I* is the identity matrix, and *C_m_* and *C_r_* are the stiffness tensors for the concrete matrix and the nano-iron reinforcement, respectively. Meanwhile, *V_m_* and *V_r_* are the volume fraction of the matrix and reinforcement, and *S* is the Eshelby tensor which is related to the Poisson ratios of nanoparticles.

For both isotropic materials, the stiffnesses *C_m_* and *C_r_* are expressed in Equation (2):

(2a) $$C_{11}=C_{22}=\frac{\left(1-\upsilon \right)E}{\left(1+\upsilon \right)\left(1-2\upsilon \right)}$$

(2b) $$C_{12}=\frac{\upsilon E}{\left(1+\upsilon \right)\left(1-2\upsilon \right)}$$

(2c) $$C_{44}=C_{55}=C_{66}=\frac{E}{\left(1+\upsilon \right)}$$

In which *E* is the Young’s modulus of either the concrete matrix or the iron nanoparticle reinforcement, and *υ* is Poisson’s ratios. The indexes 1, 2, and 3 conform to the *x*, *y*, *z* directions of the composite Cartesian co-ordinate system, respectively.

Reinforcement with spherical form *S* is given in Equation (3) (Clyne and Withers [38]):

(3) $$S=\left[\begin{array}{cccccc} S_{1111} & S_{1122} & S_{1133} & S_{1123} & S_{1113} & S_{1112}\\ S_{2211} & S_{2222} & S_{2233} & S_{2223} & S_{2213} & S_{2212}\\ S_{3311} & S_{3322} & S_{3333} & S_{3323} & S_{3313} & S_{3312}\\ S_{2311} & S_{2322} & S_{2333} & S_{2323} & S_{2313} & S_{2312}\\ S_{1311} & S_{1322} & S_{1333} & S_{1323} & S_{1313} & S_{1312}\\ S_{1211} & S_{1222} & S_{1233} & S_{1123} & S_{1213} & S_{1212} \end{array}\right]$$

where the Eshelby’s matrix components are given in Equation (4):

(4a) $$S_{1111}=S_{2222}=S_{3333}=\frac{7-5\upsilon_{r}}{15\left(1-\upsilon_{r}\right)}$$

(4b) $$S_{1122}=S_{1133}=S_{2233}=S_{2211}=S_{3311}=S_{3322}=\frac{-1+5\upsilon_{r}}{15\left(1-\upsilon_{r}\right)}$$

(4c) $$S_{1212}=S_{1313}=S_{2323}=\frac{4-5\upsilon_{r}}{15\left(1-\upsilon_{r}\right)}$$

In relation (4), *υ_r_* denotes the Poisson’s ratio of nanoparticle reinforcements.

For particulate composites, Eshelby’s approach for the prediction of the thermal expansion α_T_ is presented in Equation (5):

(5) $$\alpha_{T}=\alpha_{m}-V_{r}{\left\{\left(C_{m}-C_{r}\right)\left[S-V_{r}\left(S-I\right)\right]-C_{m}\right\}}^{-1}C_{r}\left(\alpha_{r}-\alpha_{m}\right)$$

In Equation (5), *α_r_* and *α_m_* are the reinforcement and matrix expansion tensors, respectively. *C_m_* and *C_r_* are the same stiffness tensors, but *S* tensor is now appropriate for heat properties, in which

(6) $$S_{11}=S_{22}=S_{33}=\frac{1}{3}\;{\mathrm{For}\;\mathrm{all}\;\mathrm{others}\;S}_{ij}=0$$

## 3. Mathematical Modeling of the Plate

**Figure 2:**
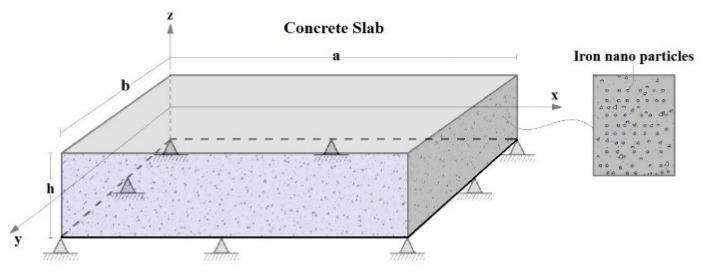
Geometry and coordinate of a simply supported nano Fe_2_O_3_-reinforced concrete.

A simply supported flat concrete slab having a length ‘*a*’, width ‘*b’*, and total thickness ‘*h*’ reinforced with iron oxide particles is considered in this study. The Fe_2_O_3_ nanoparticles are supposed to be randomly distributed in the concrete matrix as illustrated in Figure 2. The chosen coordinate system (*x*, *y*, *z*) is also shown in Figure 2, in which

(7) 0≤x≤a; 0≤y≤b;−h/2≤z≤h/2.

For comparison purposes, different higher order shear deformation theories (HSDT’s) were used in our analysis. The HSDT displacement field of a material point located at (*x*, *y*, *z*) in the plate can be described in Equation (8):

(8a) $$U\left(x,y,z\right)=u_{0}\left(x,y\right)-z\frac{\partial w_{0}\left(x,y\right)}{\partial x}+f\left(z\right)\theta_{x}$$

(8b) $$V(x,y,z)=v_{0}\left(x,y\right)-z\frac{\partial w_{0}\left(x,y\right)}{\partial y}+f\left(z\right)\theta_{y}$$

(8c) $$W(x,y,z)=w_{0}\left(x,y\right)$$

where *U*, *V*, and *W* are displacements in *x*, *y*, *z* directions; *u*_0_, *v*_0_, and *w*_0_ are mid-plane translations, and *θx* and *θy* are rotations of normal to the mid-plane around *y*-axis and *x*-axis, respectively. *f*(*z*) represents the shape function that determines the distribution of transverse shear strain and stress through the thickness. The displacement of parabolic shear deformation plate theory (PSDPT) is obtained by setting (Reddy [23])

(9) $$f\left(z\right)=z\left(1-\frac{4z^{2}}{3h^{2}}\right)$$

Additionally, the sinusoidal shear deformation plate theory (SSDPT) is obtained by setting (Touratier et al. [24])

(10) $$f\left(z\right)=\frac{h}{\pi }\sin\left(\frac{\pi z}{h}\right)$$

In addition, the displacement of exponential shear deformation plate theory (ESDPT) is obtained by setting (Karama et al. [39])

(11) $$f\left(z\right)=ze^{-2{\left(z/h\right)}^{2}}$$

### 3.1. Assumptions of the Trigonometric Refined Plate Theory (RTSDT)

Unlike other theories, the number of unknown functions included in the refined triangular shear strain theory (RTSDT) is only four, compared to five in other shear strain theories (Reddy [23], Touratier et al. [24], Karama et al. [39]). RTSDT does not require a shear correction factor, such as in the case of the first order deformation theory (Mindlin 1951), and introduces a parabolic variation of transverse shear stress through the thickness of the plate.

The main assumptions of the refined trigonometric shear deformation theory are recalled in the following:

- The displacements are small besides the plate total thickness ‘*h*’; therefore, strains involved are considered infinitesimal.
- The transverse displacement ‘*w*’ is divided into two components, including ‘*w_b_*’ and ‘*w_s_*’, accounting for the bending and shear effects; these components are the function of the spatial coordinates *x*, *y* and of time *t* only.

(12) $$W\left(x,y,t\right)=w_{b}\left(x,y,t\right)+w_{s}\left(x,y,t\right)$$

As a result of the previous assumptions, the displacements ‘*U*’ in ‘*x*’ direction and ‘*V*’ in ‘*y*’ direction can be expressed by means of the translation, bending, and shear components:

(13a) $$U=u_{0}+u_{b}+u_{s}$$

(13b) $$V=v_{0}+v_{b}+v_{s}$$

The bending components ‘*u_b_*’ and ‘*v_b_*’ are assumed to be identical to the displacements given by the classical plate theory. Therefore, the expression for ‘*u_b_*’ and ‘*v_b_*’ can be given by Equation (14):

(14a) $$u_{b}=z\frac{\partial w_{0}}{\partial x}$$

(14b) $$v_{b}=z\frac{\partial w_{0}}{\partial y}$$

The shear components ‘*u_s_*’ and ‘*v_s_*’ are defined in this theory to express the shear displacement effect ‘*w_s_*’ to different variations of shear strains *γ_xz_* and *γ_y_*_z_ and, hence, to shear stresses *σ_xz_* and *σ_yz_* across the thickness of the plate in such a way that shear stresses *σ_xz_* and *σ_yz_* are zero at the top and bottom faces of the plate. Consequently, ‘*u_s_*’ and ‘*v_s_*’ can be put as in Equation (15):

(15a) $$u_{s}=f\left(z\right)\frac{\partial w_{0}}{\partial x}$$

(15b) $$v_{s}=f\left(z\right)\frac{\partial w_{0}}{\partial y}$$

Here, ‘*f*(*z*)*’* represents the shape function determining the transverse shear distribution through the plate thickness. The transverse normal stress ‘*σ_z_*’ is assumed to be negligible in comparison with the in-plane stresses ‘*σ_x_*’ and ‘*σ_y_*’. The body force of the plate is also neglected in this analysis.

### 3.2. Kinematics

Based on Equations (12)–(15), the displacement field in the trigonometric refined theory can be written as:

(16a) $$U\left(x,y,z\right)=u_{0}\left(x,y\right)-z\frac{\partial w_{b}\left(x,y\right)}{\partial x}-f\left(z\right)\frac{\partial w_{s}\left(x,y\right)}{\partial x}$$

(16b) $$V(x,y,z)=v_{0}\left(x,y\right)-z\frac{\partial w_{b}\left(x,y\right)}{\partial y}-f\left(z\right)\frac{\partial w_{s}\left(x,y\right)}{\partial y}$$

(16c) $$W(x,y,z)=w_{b}\left(x,y\right)+w_{s}\left(x,y\right)$$

where ‘*u*_0_’ and ‘*v*_0_′ are the mid-plane displacements of the plate along the *x* and *y* direction. ‘*w*_b_*’* and ‘*w*_s_*’* are the bending and shear components of transverse displacement in *z* direction, respectively.

In this analysis, the trigonometric shape function is presented as

(17) $$f\left(z\right)=z-\left(\frac{h}{\pi }\right)\sin\left(\frac{\pi }{h}z\right)$$

It should be noted that, unlike the first-order shear deformation theory, refined plate theories do not require shear correction factors.

The linear strains components can be derived from the displacement field in Equation (16) and are given in Equation (18):

(18a) $$\varepsilon_{x}=\varepsilon_{x}^{0}-zk_{x}^{b}-f\left(z\right)k_{x}^{s}$$

(18b) $$\gamma_{xy}=\gamma_{xy}^{0}-z\gamma_{xy}^{b}-f\left(z\right)\gamma_{xy}^{s}$$

(18c) $$\varepsilon_{y}=\varepsilon_{y}^{0}-zk_{y}^{b}-f\left(z\right)k_{y}^{s}$$

(18d) $$\gamma_{yz}=\left(1-\frac{\partial f\left(z\right)}{\partial z}\right)\gamma_{yz}^{s}=g\left(z\right)\gamma_{yz}^{s}$$

(18e) $$\varepsilon_{z}=0$$

(18f) $$\gamma_{xz}=\left(1-\frac{\partial f\left(z\right)}{\partial z}\right)\gamma_{xz}^{s}=g\left(z\right)\gamma_{xz}^{s}$$

where

(19a) $$\varepsilon_{x}^{0}=\frac{\partial u_{0}}{\partial x}$$

(19b) $$k_{x}^{b}=\frac{\partial w_{b}}{\partial x}$$

(19c) $$k_{x}^{s}=\frac{\partial w_{s}}{\partial x}$$

(19d) $$\varepsilon_{y}^{0}=\frac{\partial v_{0}}{\partial y}$$

(19e) $$k_{x}^{b}=\frac{\partial w_{b}}{\partial y}$$

(19f) $$k_{x}^{s}=\frac{\partial w_{s}}{\partial y}$$

(19g) $$\gamma_{xy}^{0}=\frac{\partial v_{0}}{\partial x}+\frac{\partial u_{0}}{\partial y}$$

(19h) $$k_{x}^{b}=-2\frac{\partial^{2}w_{b}}{\partial x\partial y}$$

(19i) $$k_{x}^{s}=\frac{\partial^{2}w_{s}}{\partial x\partial y}$$

(19j) $$\gamma_{yz}^{s}=\frac{\partial w_{s}}{\partial y}$$

(19k) $$\gamma_{xz}^{s}=\frac{\partial w_{s}}{\partial x}$$

(19l) $$g\left(z\right)=1-\frac{\partial f\left(z\right)}{\partial z}$$

The constitutive stress–strain relations of the nano-composite can be defined as

(20) $$\begin{array}{c} \left\{\begin{array}{l} \sigma_{x}\\ \sigma_{y}\\ \tau_{xy}\\ \tau_{yz}\\ \tau_{xz} \end{array}\right\}=\left[\begin{array}{lllll} C_{T}^{11} & C_{T}^{12} & 0 & 0 & 0\\ C_{T}^{12} & C_{T}^{22} & 0 & 0 & 0\\ 0 & 0 & C_{T}^{55} & 0 & 0\\ 0 & 0 & 0 & C_{T}^{44} & 0\\ 0 & 0 & 0 & 0 & C_{T}^{66} \end{array}\right]\left\{\begin{array}{l} \varepsilon_{x}-\alpha T\\ \varepsilon_{y}-\alpha T\\ \gamma_{xy}\\ \gamma_{yz}\\ \gamma_{xz} \end{array}\right\} \end{array}$$

where $C_{T}^{ij}$ are the reduced elastic constants of the concrete slab reinforced with Fe_2_O_3_, obtained using Eshelby’s homogenization model.

### 3.3. Principle of Virtual Work

The virtual work principle is applied to develop the equations of motion in the present case yields:

(21) $$\int_{0}^{t}\left(\delta \Psi +\delta \Phi \right)\partial t=0$$

where *δΨ* and *δΦ* are the virtual variation of the internal strain energy and the virtual work performed by external forces, respectively.

The expression of the virtual strain energy is

(22) $$\delta \Psi =\int_{-h/2}^{h/2}\int_{A}\left(\sigma_{x}\delta \varepsilon_{x}+\sigma_{y}\delta \varepsilon_{y}+\tau_{xy}\delta \gamma_{xy}+\tau_{yz}\delta \gamma_{yz}+\tau_{xz}\delta \gamma_{xz}\right)\partial A\partial z$$

By substituting Equation (18) into Equation (22), one finds

(23) $$\delta \Psi =\int_{A}\left\{\begin{array}{l} N_{x}\delta u_{0,x}-M_{x}^{b}\delta w_{b,x}+M_{x}^{s}\delta w_{s,x}+N_{y}\delta v_{0,y}-M_{y}^{b}\delta w_{b,x}\\ +M_{y}^{s}\delta w_{s,x}+N_{xy}\left(\delta u_{0,y}+\delta v_{0,x}\right)+M_{xy}^{b}2\delta w_{b,x,y}\\ +M_{xy}^{s}2\delta w_{s,x,y}+Q_{yz}\delta w_{s,y}+Q_{xz}\delta w_{s,x} \end{array}\right\}\partial A$$

where stress resultants can be expressed in Equation (24):

(24a) $$\left(N_{x},N_{y},N_{xy}\right)=\int_{-h/2}^{h/2}\left(\sigma_{x},\sigma_{y},\tau_{xy}\right)$$

(24b) $$\left(M_{x}^{b},M_{y}^{b},M_{xy}^{b}\right)=\int_{-h/2}^{h/2}z\left(\sigma_{x},\sigma_{y},\tau_{xy}\right)$$

(24c) $$\left(M_{x}^{s},M_{y}^{s},M_{xy}^{s}\right)=\int_{-h/2}^{h/2}f\left(z\right)\left(\sigma_{x},\sigma_{y},\tau_{xy}\right)$$

(24d) $$\left(Q_{yz},Q_{xz}\right)=\int_{-h/2}^{h/2}g\left(z\right)\left(\sigma_{yz},\sigma_{xz}\right)$$

For the concrete plates subjected to bending loads ‘‘*q*’’, the virtual work performed by external loadings is

(25) $$\delta \Phi =-\int_{0}^{A}q\left(x,y\right)\left(\delta w_{b}+\delta w_{s}\right)\partial x\partial y$$

The equilibrium equations associated with the trigonometric refined shear deformation theory can be derived from Equation (21) by integrating parts and collecting the coefficients of *δu*_0_, *δv*_0_, *δw_b_*, and *δw_s_* and are given in Equation (26):

(26a) $$\delta u_{0}\;:\;\frac{\partial N_{x}}{\partial x}+\frac{\partial N_{xy}}{\partial y}=0$$

(26b) $$\delta v_{0}\;:\;\frac{\partial N_{y}}{\partial y}+\frac{\partial N_{xy}}{\partial x}=0$$

(26c) $$\delta w_{b}\;:\;\frac{\partial^{2}M_{x}^{b}}{\partial x^{2}}+\frac{\partial^{2}M_{xy}^{b}}{\partial xdy}+\frac{\partial^{2}M_{x}^{b}}{\partial y^{2}}+q=0$$

(26d) $$\delta w_{s}\;:\;\frac{\partial^{2}M_{x}^{s}}{\partial x^{2}}+\frac{\partial^{2}M_{xy}^{s}}{\partial xdy}+\frac{\partial^{2}M_{x}^{s}}{\partial y^{2}}+\frac{\partial Q_{yz}}{\partial y}+\frac{\partial Q_{xz}}{\partial x}+q=0$$

By substituting Equation (20) into Equation (24), one obtains the stress resultants in form of material stiffness and displacement components:

(27a) $$\left\{\begin{array}{l} N_{x}\\ N_{y}\\ N_{xy} \end{array}\right\}=\left[\begin{array}{ccc} A_{11} & A_{12} & 0\\ A_{12} & A_{22} & 0\\ 0 & 0 & A_{66} \end{array}\right]\left\{\begin{array}{l} \frac{\partial u_{0}}{\partial x}\\ \frac{\partial v_{0}}{\partial y}\\ \frac{\partial u_{0}}{\partial y}+\partial \frac{\partial v_{0}}{\partial x} \end{array}\right\}\;+\left[\begin{array}{ccc} B_{11} & B_{12} & 0\\ B_{12} & B_{22} & 0\\ 0 & 0 & B_{66} \end{array}\right]\left\{\begin{array}{l} \frac{\partial^{2}w_{b}}{\partial x^{2}}\\ \partial \frac{\partial^{2}w_{b}}{dy^{2}}\\ -2\frac{\partial^{2}w_{b}}{\partial x\partial y} \end{array}\right\}+\left[\begin{array}{ccc} Bs_{11} & Bs_{12} & 0\\ Bs_{12} & Bs_{22} & 0\\ 0 & 0 & Bs_{66} \end{array}\right]\left\{\begin{array}{l} \frac{\partial^{2}w_{s}}{\partial x^{2}}\\ \frac{\partial^{2}w_{s}}{\partial y^{2}}\\ -2\frac{\partial^{2}w_{s}}{\partial x\partial y} \end{array}\right\}\;-\left\{\begin{array}{l} N_{T}^{x}\\ N_{T}^{y}\\ N_{T}^{xy} \end{array}\right\}$$

(27b) $$\left\{\begin{array}{l} M_{x}^{b}\\ M_{y}^{b}\\ M_{xy}^{b} \end{array}\right\}=\left[\begin{array}{ccc} B_{11} & B_{12} & 0\\ B_{12} & B_{22} & 0\\ 0 & 0 & B_{66} \end{array}\right]\left\{\begin{array}{l} \frac{\partial u_{0}}{\partial x}\\ \frac{\partial v_{0}}{\partial y}\\ \frac{\partial u_{0}}{\partial y}+\frac{\partial v_{0}}{\partial x} \end{array}\right\}+\left[\begin{array}{ccc} D_{11} & D_{12} & 0\\ D_{12} & D_{22} & 0\\ 0 & 0 & D_{66} \end{array}\right]\left\{\begin{array}{l} \partial \frac{\partial^{2}w_{b}}{dx^{2}}\\ \frac{\partial^{2}w_{b}}{\partial y^{2}}\\ -2\frac{\partial^{2}w_{b}}{\partial x\partial y} \end{array}\right\}\;+\left[\begin{array}{ccc} Bs_{11} & Bs_{12} & 0\\ Bs_{12} & Bs_{22} & 0\\ 0 & 0 & Bs_{66} \end{array}\right]\left\{\begin{array}{l} \frac{\partial^{2}w_{s}}{\partial x^{2}}\\ \frac{\partial^{2}w_{s}}{\partial y^{2}}\\ -2\frac{\partial^{2}w_{s}}{\partial x\partial y} \end{array}\right\}-\left\{\begin{array}{l} N_{T}^{x}\\ N_{T}^{y}\\ N_{T}^{xy} \end{array}\right\}$$

(27c) $$\left\{\begin{array}{l} M_{x}^{s}\\ M_{y}^{s}\\ M_{xy}^{s} \end{array}\right\}=\left[\begin{array}{ccc} Bs_{11} & Bs_{12} & 0\\ Bs_{12} & Bs_{22} & 0\\ 0 & 0 & Bs_{66} \end{array}\right]\left\{\begin{array}{l} \frac{\partial u_{0}}{\partial x}\\ \frac{\partial v_{0}}{\partial y}\\ \frac{\partial u_{0}}{\partial y}+\frac{\partial v_{0}}{\partial x} \end{array}\right\}\;+\left[\begin{array}{ccc} Ds_{11} & Ds_{12} & 0\\ Ds_{12} & Ds_{22} & 0\\ 0 & 0 & Ds_{66} \end{array}\right]\left\{\begin{array}{l} \frac{\partial^{2}w_{b}}{\partial x^{2}}\\ \frac{\partial^{2}w_{b}}{\partial y^{2}}\\ -2\frac{\partial^{2}w_{b}}{\partial x\partial y} \end{array}\right\}+\left[\begin{array}{ccc} Hs_{11} & Hs_{12} & 0\\ Hs_{12} & Hs_{22} & 0\\ 0 & 0 & Hs_{66} \end{array}\right]\left\{\begin{array}{l} \frac{\partial^{2}w_{s}}{\partial x^{2}}\\ \frac{\partial^{2}w_{s}}{\partial y^{2}}\\ -2\frac{\partial^{2}w_{s}}{\partial x\partial y} \end{array}\right\}\;-\left\{\begin{array}{l} M_{sT}^{x}\\ M_{sT}^{y}\\ M_{sT}^{xy} \end{array}\right\}$$

(27d) $$\left\{\begin{array}{c} Q_{yz}\\ Q_{xz} \end{array}\right\}=\left[\begin{array}{cc} As_{44} & 0\\ 0 & As_{55} \end{array}\right]\left\{\begin{array}{c} \frac{\partial w_{s}}{\partial y}\\ \frac{\partial w_{s}}{\partial x} \end{array}\right\}$$

where *A_ij_*, *B_ij_*, *D_ij_*, *Bs_ij_*, *Ds_ij_*, *Hs_ij_*, and *As_ij_* are the plate stiffness, defined in Equation (28):

(28a) $$\left[A_{ij},B_{ij},D_{ij}\right]=\int_{h_{n}}^{h_{n+1}}C_{ij}\left[1,z,z^{2}\right]\partial z;$$

(28b) $$\left[Bs_{ij},Ds_{ij},Hs_{ij}\right]=\int_{h_{n}}^{h_{n+1}}C_{ij}\left[f\left(z\right),zf\left(z\right),f{\left(z\right)}^{2}\right]\partial z;$$

(28c) $$\left[As_{ij}\right]=\int_{h_{n}}^{h_{n+1}}C_{ij}\left[g{\left(z\right)}^{2}\right]\partial z.$$

The normal stress and moment resultants $N_{T}^{x}=N_{T}^{y}$,$M_{bT}^{x}=M_{bT}^{y}$, and $M_{sT}^{x}=M_{sT}^{y}$ caused by the external thermal loading are defined by

(29) $$\left\{\begin{array}{l} N_{T}^{x}\\ M_{bT}^{x}\\ M_{ST}^{x} \end{array}\right\}=\int_{-h/2}^{h/2}C_{T}^{11}\alpha_{T}T\left\{\begin{array}{l} 1\\ z\\ f\left(z\right) \end{array}\right\}$$

It is assumed that the variation of the temperature field through the thickness is expressed as

(30) $$T\left(x,y,z\right)=T_{1}\left(x,y\right)+\frac{z}{h}T_{2}\left(x,y\right)+\frac{\psi \left(z\right)}{h}T_{3}\left(x,y\right)$$

Here, *T*_1_, *T*_2_ and *T*_3_ are thermal loadings, and for the case of the present refined trigonometric theory (RTSDT),

(31) $$\psi \left(z\right)=\frac{h}{\pi }sin\left(\frac{\pi z}{h}\right)$$

Substituting from Equation (27) into Equation (26), the following equations are obtained:

(32a) $$\left\{\begin{array}{l} A_{11}d_{11}u_{0}{+A}_{66}d_{22}u_{0}+\left(A_{12\;}{+A}_{66}\right)d_{12}v_{0}{\;-\;B}_{11}d_{111}w_{b}\\ -\;\left(B_{12}{+2B}_{66}\right)d_{122}w_{b\;}\\ -\;\left(B_{s12}{+2B}_{s66}\right)d_{122}w_{s\;}{-\;B}_{s11}d_{111}w_{s}=f_{1} \end{array}\right\}$$

(32b) $$\left\{\begin{array}{l} A_{22}d_{22}v_{0}{+A}_{66}d_{22}v_{0}+\left(A_{12\;}{+A}_{66}\right)d_{12}u_{0}{\;-\;B}_{22}d_{222}w_{b}\\ -\;\left(B_{12}{+2B}_{66}\right)d_{112}w_{b\;}\\ -\;\left(B_{s12}{+2B}_{s66}\right)d_{112}w_{s\;}{-\;B}_{s22}d_{222}w_{s}=f_{2} \end{array}\right\}$$

(32c) $$\left\{\begin{array}{l} B_{11}d_{111}u_{0}+\left(B_{12\;}{+B}_{66}\right)d_{122}u_{0}+\left(B_{12\;}{+B}_{66}\right)d_{112}v_{0}\\ +B_{22}d_{222}v_{0}{-D}_{11}d_{1111}w_{b\;}\\ -\;2\left(D_{12}{+2D}_{66}\right)d_{1122}w_{b\;}{-D}_{22}d_{2222}w_{b\;}{-D}_{s11}d_{1111}w_{s\;}\\ -\;2\left(D_{s12}{+2D}_{s66}\right)d_{1122}w_{s\;}{-D}_{s22}d_{2222}w_{s\;}=f_{3} \end{array}\right\}$$

(32d) $$\left\{\begin{array}{l} B_{s11}d_{111}u_{0}+\left(B_{s12}+B_{s66}\right)d_{122}u_{0}+\left(B_{s12}+B_{s66}\right)d_{112}v_{0}\\ +B_{s22}d_{222}v_{0}-D_{s11}d_{1111}w_{b}-2\left(D_{s12}+2D_{s66}\right)d_{1122}w_{b}\\ -D_{s22}d_{2222}w_{b}-D_{s11}d_{1111}w_{s}-2\left(H_{s12}+2H_{s66}\right)d_{1122}w_{s}\\ -H_{s22}d_{2222}w_{s}-A_{s55}d_{11}w_{s}-A_{s44}d_{22}w_{s}=f_{4} \end{array}\right\}$$

The generalized force vector is described as {*f*} = {*f*_1_, *f*_2_, *f*_3_, *f*_4_}*^T^*, while *d_i j_*, *d_i jl_*, and *d_i jlm_* are differential operators expressed as the following:

(33a) $$\mathrm{For}\;\left(i,j,l,m=1,2\right):\;d_{i}=\frac{\partial }{\partial x_{i}}$$

(33b) $$d_{ij}=\frac{\partial^{2}}{\partial x_{i}\partial x_{j}}$$

(33c) $$d_{ijl}=\frac{\partial^{3}}{\partial x_{i}\partial x_{j}\partial x_{l}}$$

(33d) $$d_{ijlm}=\frac{\partial^{4}}{\partial x_{i}\partial x_{j}\partial x_{l}\partial x_{m}}$$

The components of the generalized force vector {*f*} are

(34a) $$f_{1}=\frac{\partial N_{T}^{x}}{\partial x}$$

(34b) $$f_{2}=\frac{\partial N_{T}^{y}}{\partial y}$$

(34c) $$f_{3}=-\frac{\partial^{2}M_{bT}^{x}}{\partial x^{2}}-\frac{\partial^{2}M_{bT}^{y}}{\partial y^{2}}$$

(34d) $$f_{4}=-\frac{\partial^{2}M_{sT}^{x}}{\partial x^{2}}-\frac{\partial^{2}M_{sT}^{y}}{\partial y^{2}}$$

### 3.4. Navier’s Technique

To formulate the closed-form solutions for bending problems of simply supported rectangular plates for the refined trigonometric shear deformation theory, Navier’s method is employed:

(35a) $$u_{0}\left(x,y,t\right)=\overset{\infty }{\underset{m=1}{\sum }}\overset{\infty }{\underset{n=1}{\sum }}U_{mn}cos\left(\alpha x\right)sin\left(\varphi y\right)$$

(35b) $$v_{0}\left(x,y,t\right)=\overset{\infty }{\underset{m=1}{\sum }}\overset{\infty }{\underset{n=1}{\sum }}V_{mn}sin\left(\alpha x\right)cos\left(\varphi y\right)$$

(35c) $$w_{b}\left(x,y,t\right)=\overset{\infty }{\underset{m=1}{\sum }}\overset{\infty }{\underset{n=1}{\sum }}W_{bmn}sin\left(\alpha x\right)sin\left(\varphi y\right)$$

(35d) $$w_{s}\left(x,y,t\right)=\overset{\infty }{\underset{m=1}{\sum }}\overset{\infty }{\underset{n=1}{\sum }}W_{smn}sin\left(\alpha x\right)sin\left(\varphi y\right)$$

where *U_mn_*, *V_mn_*, *W_bmn_*, and *W_smn_* are the arbitrary parameters to be determined, α = *mπ/a*, and φ = *nπ/b*.

The transverse load ‘*q*’ for the mechanical bending is expressed by the double-Fourier sine series by

(36) $$q\left(x,y\right)=\overset{\infty }{\underset{m=1}{\sum }}\overset{\infty }{\underset{n=1}{\sum }}Q_{mn}sin\left(\alpha x\right)sin\left(\varphi y\right)$$

**Figure 3:**
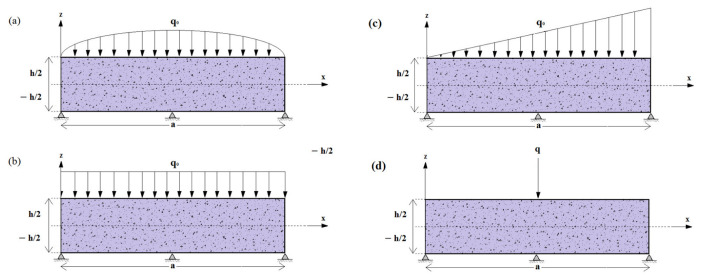
Iron oxide nanoparticles-reinforced concrete slab subjected to different mechanical bending loads; (**a**) Single sine distributed load; (**b**) Uniformly distributed load; (**c**) Linearly distributed load; (**d**) Concentrated load.

The various load patterns are illustrated in Figure 3, where the coefficients *Q_mn_* are presented below:

(37) $$Q_{mn}=\{\begin{array}{c} q_{0}\;:For\;sinsoidally\;distributed\;load\\ \frac{16q_{0}}{mn\pi^{2}}\;:For\;uniformly\;distributed\;load\\ -\frac{8q_{0}}{mn\pi^{2}}cos\left(m\pi \right)\;:For\;linearly\;distributed\;load\\ \frac{4q_{0}}{ab}sin\left(\frac{m\pi x_{0}}{a}\right)sin\left(\frac{m\pi y_{0}}{b}\right)\;:For\;concentrated\;load \end{array}$$

Navier presented the transverse temperature loads *T*_1_, *T*_2_, and *T*_3_ in the form of a double trigonometric series as

(38) $$\left\{\begin{array}{l} T_{1}\\ T_{2}\\ T_{3} \end{array}\right\}=\left\{\begin{array}{l} {\bar{T}}_{1}\\ {\bar{T}}_{2}\\ {\bar{T}}_{3} \end{array}\right\}sin\left(\alpha x\right)sin\left(\varphi y\right).$$

Substituting Equation (35) into Equation (26), one obtains the closed-form solutions in matrix form:

(39) $$\left[K_{ij}\right]\left\{\Delta \right\}=\left\{f\right\}$$

where

(40) $$\left\{\Delta \right\}={\left\{U_{mn},V_{mn},W_{bmn},W_{smn}\right\}}^{T}$$

The components of the elastic stiffness matrix [*K*_ij_] are as follows:

(41) $$\left[K_{ij}\right]=\left[\begin{array}{cccc} k_{11} & k_{12} & k_{13} & k_{14}\\ k_{21} & k_{22} & k_{23} & k_{24}\\ k_{31} & k_{32} & k_{33} & k_{34}\\ k_{41} & k_{42} & k_{43} & k_{44} \end{array}\right]$$

where the elements of the stiffness matrix are given in Equation (42):

(42) $$k_{11}=-A_{11}\alpha^{2}-A_{66}\varphi^{2};{\;k}_{12}=-\alpha \varphi \left(A_{11}+A_{66}\right);\;k_{13}=B_{11}\alpha^{3}+B_{12}\alpha \varphi^{2}+2B_{66}\alpha \varphi^{2};\;\;k_{14}=Bs_{11}\alpha^{3}+Bs_{12}\alpha \varphi^{2}+2Bs_{66}\alpha \varphi^{2};\;\;k_{21}=k_{12};\begin{array}{c} k_{22}=-A_{66}\alpha^{2}-A_{22}\varphi^{2}; \end{array}\;k_{23}=B_{11}\alpha^{3}+B_{12}\varphi \alpha^{2}+2B_{66}\varphi \alpha^{2}\;k_{24}=Bs_{11}\alpha^{3}+Bs_{12}\varphi \alpha^{2}+2Bs_{66}\varphi \alpha^{2}\;k_{31}=k_{13};{\;k}_{32}=k_{23};\;\;k_{33}=-D_{11}\alpha^{4}-2\alpha^{2}\varphi^{2}\left(D_{12}+2D_{66}\right)-D_{22}\varphi^{4}\;k_{34}=-Ds_{11}\alpha^{4}-2\alpha^{2}\varphi^{2}\left(Ds_{12}+2Ds_{66}\right)-Ds_{22}\varphi^{4};\;k_{41}=k_{14};{\;k}_{42}=k_{24};{\;k}_{43}=k_{34}\;k_{44}=-Hs_{11}\alpha^{4}-2\alpha^{2}\varphi^{2}\left(D_{12}+2D_{66}\right)-Ds_{22}\varphi^{4}-As_{44}\alpha^{2}-As_{55}\alpha^{2}$$

The components of the generalized thermal force vector {*f*} = {*f*_1_, *f*_2_, *f*_3_, *f*_4_}*^T^* are given by

(43a) $$f_{1}=\alpha \left(A_{T}\bar{T_{1}}+B_{T}\bar{T_{2}}+B_{T}^{a}\bar{T_{3}}\right)$$

(43b) $$f_{2}=\varphi \left(A_{T}\bar{T_{1}}+B_{T}\bar{T_{2}}+B_{T}^{a}\bar{T_{3}}\right)$$

(43c) $$f_{3}=-h\left(\alpha^{2}+\varphi^{2}\right)\left(B_{T}\bar{T_{1}}+D_{T}\bar{T_{2}}+D_{T}^{a}\bar{T_{3}}\right)$$

(43d) $$f_{4}=-h\left(\alpha^{2}+\varphi^{2}\right)\left(B_{T}^{s}\bar{T_{1}}+D_{T}^{s}\bar{T_{2}}+F_{T}^{s}\bar{T_{3}}\right)$$

where

(44) $$\left[A_{T},B_{T},D_{T}\right]=\int_{-h/2}^{h/2}C_{T}^{11}\left(1+\upsilon_{T}\right)\alpha_{T}\left[1,\bar{z},{\bar{z}}^{2}\right]\partial z\;\left[B_{T}^{a},D_{T}^{a}\right]=\int_{-h/2}^{h/2}C_{T}^{11}\left(1+\upsilon_{T}\right)\alpha_{T}\bar{\psi }\left(z\right)\left[1,\bar{z}\right]\partial z\;\left[B_{T}^{s},D_{T}^{s},F_{T}^{s}\right]=\int_{-h/2}^{h/2}C_{T}^{11}\left(1+\upsilon_{T}\right)\alpha_{T}\bar{f}\left(z\right)\left[1,\bar{z},\bar{\psi }\left(z\right)\right]\partial z$$

in which

(45) $$\bar{z}=z/h,\;\;\;\;\bar{f}\left(z\right)=f\left(z\right)/h,\;\;\;\;and\;\;\;\;\bar{\psi }\left(z\right)=\psi \left(z\right)/h$$

## 4. Results and Discussion

To analyze the static behavior of simply supported nano-iron-reinforced concrete slabs, various numerical examples are presented and discussed to predict the mechanical and thermoelastic bending of the slab by using the refined trigonometric shear deformation theory (RTSDT).

Herein is presented a concrete slab with Young’s modulus of *E_m_* = 20 GPa and thermal expansion *α_m_* = 13.5 (10^−6^/K). The concrete matrix is incorporated with iron nanoparticles for Young’s modulus of *E_r_* = 200 GPa and thermal expansion *α_r_* = 12 (10^−6^/K). Poisson’s ratios are *υ_m_* = 0.3 and *υ_r_* = 0.291 for concrete and iron nanoparticles, respectively. Dimensionless displacements and stresses generated by the mechanical external loads are presented according to the following definitions:

(46a) $$\sigma_{x}\left(z\right)=-\frac{h}{q_{0}L}\sigma_{x}\left(\frac{L}{2},\frac{b}{2},z\right)\;\;\;\;\;\;\;\;\;$$

(46b) $$\sigma_{xy}\left(z\right)=\frac{h}{q_{0}L}\sigma_{xy}\left(0,0,z\right)$$

(46c) $$\tau_{xz}\left(z\right)=\frac{h}{q_{0}L}\sigma_{xz}\left(0,\frac{b}{2},z\right)$$

(46d) $$\bar{U}=\frac{10E_{m}h^{3}}{q_{0}L^{4}}U\left(0,\frac{b}{2},z\right)$$

(46e) $$\omega =\frac{10E_{m}h^{3}}{q_{0}L^{4}}w\left(\frac{L}{2},\frac{b}{2},0\right)\;\;$$

Dimensionless displacements and stresses generated by the temperature loadings are expressed by the following definitions:

(47a) $$\bar{\sigma_{x}}\left(z\right)=\frac{h^{2}}{\alpha_{0}\bar{T_{2}}E_{0}a^{2}}\sigma_{x}\left(\frac{L}{2},\frac{b}{2},z\right)\;\;\;\;\;\;\;\;\;$$

(47b) $$\bar{\tau_{xz}}\left(z\right)=\frac{10h}{\alpha_{0}\bar{T_{2}}E_{0}a}\sigma_{xz}\left(0,\frac{b}{2},z\right)$$

(47c) $$\omega_{T}=\frac{h}{\alpha_{0}\bar{T_{2}}a^{2}}w\left(\frac{a}{2},\frac{b}{2},0\right)$$

To demonstrate the preceding thermal–structural analysis, different sample problems are considered. For the sake of brevity, only linearly varying (across the thickness) temperature distribution *T* = *zT*_2_, non-linearly varying (across the thickness) temperature distribution *T* = *ψ(z)T*_3_, and a combination of both *T* = *zT*_2_ + *ψ(z)T*_3_ are treated, where the reference values are *E_0_* = 1 GPa and *α*_0_ = 10^−6^/K. Note that in most of the literature, the thermal stress problems are considered under a steady state temperature distribution that is linear with respect to the thickness direction.

### 4.1. Validation

First, it is decisive to verify the adequacy of the present adopted (RTSDT) mathematical model since analytical results for the mechanical bending analysis of concrete slabs reinforced with iron nanoparticles are not available in the literature. Considering the material and geometric parameters similar to Thai and Choi [40], the results in terms of transverse displacements (ω) as well as normal stresses ($\sigma_{x},\;\sigma_{xy}$) of functionally graded (FG) slabs while varying the power index ‘*P*’ are embraced for comparison with the present refined trigonometric shear deformation theory as presented in Table 1.

As shown in Table 1, the results are generally in fair agreement. Using different shear deformation theories or shape functions gives similar results of displacements and normal stresses. However, the refined trigonometric shear deformation theory (RTSDT) predicts almost the same results as Zenkour’s refined sinusoidal shear deformation plate theory (RSSDPT).

In order to validate the elastic properties estimated by the Eshelby analytical model of a concrete matrix reinforced with ferric oxide (Fe_2_O_3_) nanoparticles, and in the absence of studies in the literature dealing with a similar analytical model, a comparison was made between the elastic stiffnesses (*C_ij_*) obtained from a concrete slab reinforced with iron nanoparticles (using the Eshelby homogenization model) and the elastic stiffnesses (*C_ij_*) obtained from a concrete slab reinforced with silica nanoparticles using the Voigt homogenization model without taking into account the agglomeration effect of (SiO_2_) nanoparticles in the concrete matrix.

**Figure 4:**
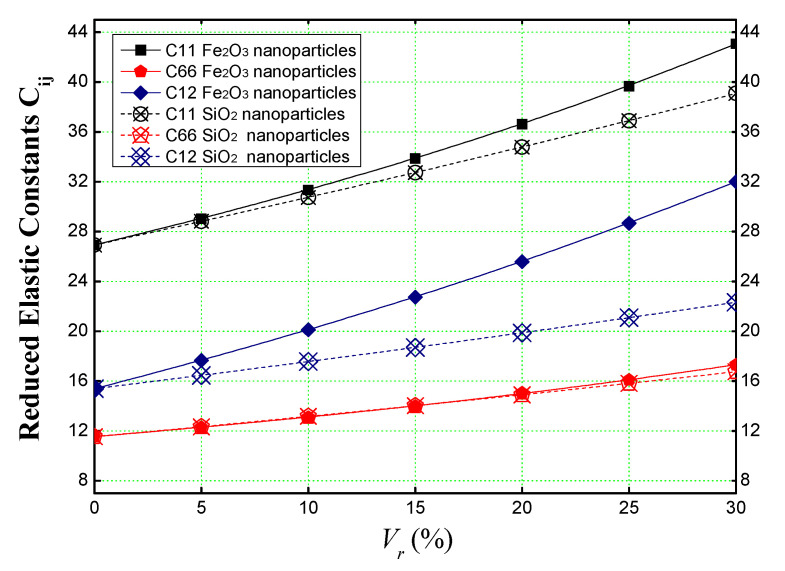
Comparison between the elastic stiffness of a concrete slab reinforced with iron/silica nanoparticles obtained by the Eshelby and Voigt models respectively.

Figure 4 shows that both types of reinforcement (Fe_2_O_3_ and SiO_2_) have the same effect on the concrete, in which the reduced elastic stiffnesses (*C_ij_*) of a concrete slab increase with the concentration of reinforcements (*V_r_*). Yet, it is especially noticeable in Figure 4 that, for iron nanoparticles (Fe_2_O_3_) reinforcements, the stiffnesses are further more improved compared to nano-silica (SiO_2_) reinforcements, particularly in the case of (*C*_12_) which denotes the elastic stiffness of the plate in (*x*, *y*) plan. This improvement is mainly due to the nanoparticle’s high mechanical properties such as Young’s modulus.

### 4.2. Part I: Bending Analysis

**Figure 5:**
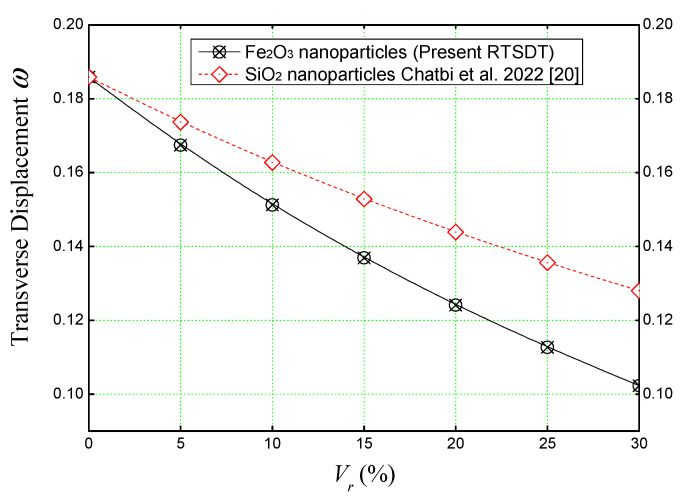
Comparison between the effect of nanoparticle reinforcements on the non-dimensional transverse displacement of concrete slabs under sinusoidal load (*a/h* = 10, *a* = *b*).

To begin with, a comparison between the non-dimensional transverse displacement values obtained using the refined trigonometric theory (RTSDT) is shown in Figure 5 as a function of reinforcement volumes. One of the curves expresses the development of the non-dimensional deflection (*ω*) of a concrete slab reinforced with iron nanoparticles Fe_2_O_3_. These material properties are determined by Eshelby’s homogenization model, and the other one expresses the variation of (*ω*) as well but this time of a concrete slab reinforced by silica nanoparticles, on which these elastic properties are determined by the Voigt homogenization law without considering the agglomeration effect of the SiO_2_ nanoparticles (Harrat et al., 2021 [19]).

It is clear from Figure 5 that the reinforcement by iron nanoparticles is more effective than by nano-silica, and this is probably due to the high physical properties of the nano-iron (Young’s elastic modulus, precisely), which leads to a higher stiffness of the concrete nano-composite.

Thus, we should retain from Figure 5 that when the plate is reinforced by nano-iron volumes, it gives us a lower deflection than a plate reinforced by SiO_2_. It is also worth noting that the higher the percentage of particles is, the lower the deflection is.

**Figure 6:**
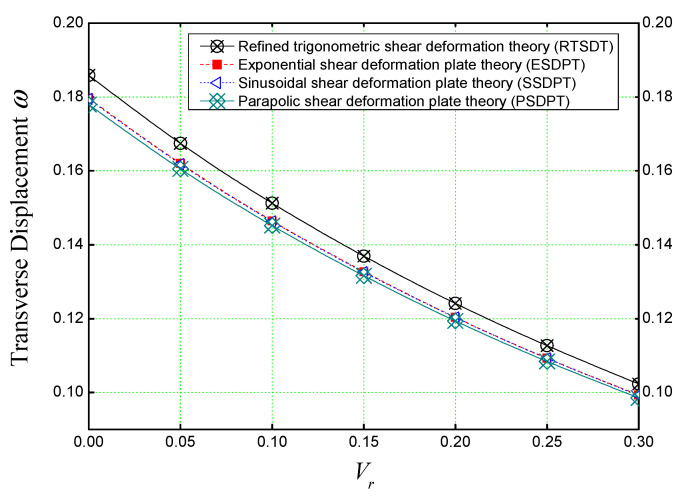
The non-dimensional deflection of a concrete slab reinforced with different volumes of Fe_2_O_3_ nanoparticles subjected to single sine load (*a/h* = 10, *a* = *b*).

Figure 6 shows the variation of the non-dimensional transverse displacement of a concrete plate reinforced with different volumes (*V_r_*) of iron nanoparticles. Several theories are used to determine the accuracy of the theory used in our analysis (the refined trigonometric shear deformation theory) in predicting the mechanical behaviors of concrete nano-composite.

From Figure 6, it should be noted that all the theories have the same behavior, but the (RTSDT) theory slightly overestimates the deflection compared to the others, and this is due to the used shape function that expresses the transverse shear stresses that evolve through the thickness of the plate.

**Figure 7:**
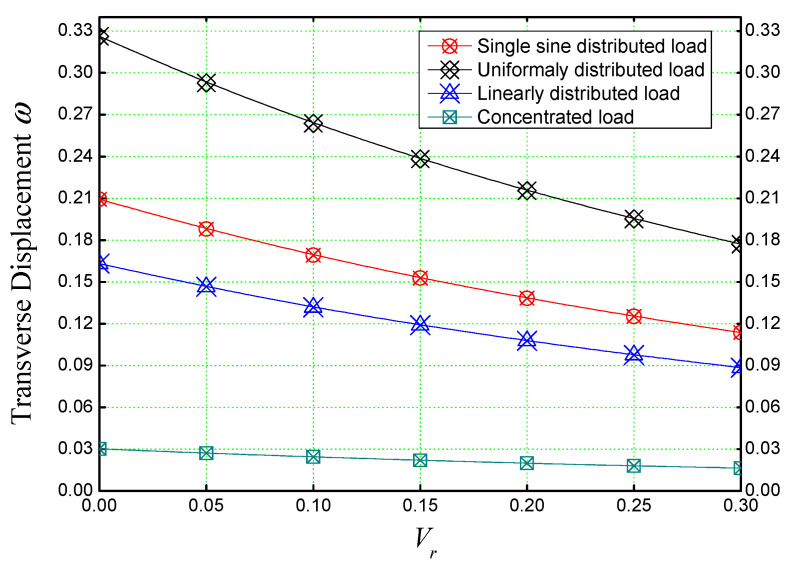
The effect of different load patterns on the non-dimensional deflection of concrete slabs reinforced with iron nanoparticles (*a/h *= 5, *a* = *b*).

Different subjected loading patterns on a rectangular (*a = b*), concrete slab reinforced with different concentrations of iron nanoparticles (Fe_2_O_3_) are shown in Figure 7.

The slab is considered simply supported (Figure 2). As it was noticed in the previous figures, the reinforcement of concrete slabs with iron nanoparticles has a strengthening role on the structure, but from Figure 7, we can say that this is actual regardless of the type of loading. It should also be noted that uniform loading has a greater effect on the bending of reinforced plates than other types of loading, while concentrated loading has the least effect.

**Figure 8:**
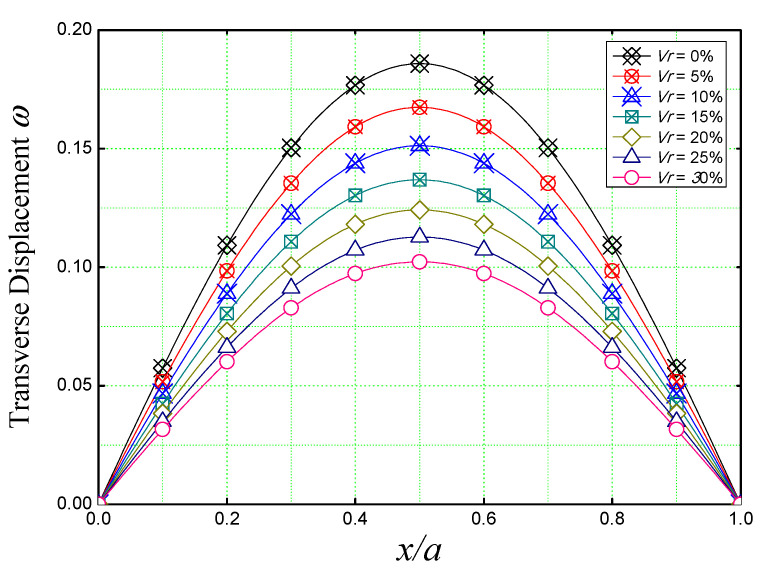
Dimensionless transverse displacement of reinforced concrete plate under sinusoidal load for various nano Fe_2_O_3_ concentrations (*a/h* = 10, *a* = *b*).

Figure 8 is used to discuss the non-dimensional transverse displacement (ω) of a simply supported square concrete plate reinforced with iron nanoparticles over the entire length *(x*/*a)* of the plate by varying the reinforcement volume from 0% (non-reinforced concrete plate) to *V_r_* = 30%. The plate is supposed to be subjected to sinusoidal loading.

Figure 8, shows that the higher deflections are obtained in the mid-length of the plate for all nano-Fe_2_O_3_ concentrations. It also confirms the stiffening effect caused by iron nanoparticles by decreasing the deflection of the nano-composite plate, knowing that the deflection of a reinforced plate (*V_r_* = 30%) is way lower (almost by half) than the non-reinforced concrete plate (*V_r_* = 0%).

**Figure 9:**
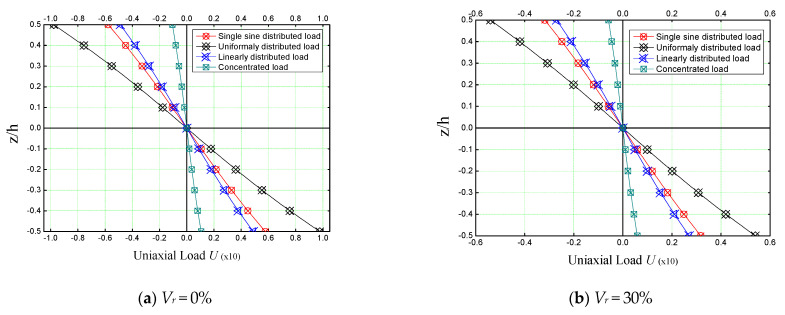
Dimensionless axial displacement of reinforced concrete square plate under various type of loads for different nano Fe_2_O_3_ concentrations (*a/h* = 10, *a* = *b*).

Figure 9 shows the variation of the non-dimensional axial displacement (*U*) of a non-reinforced concrete plate (Figure 9a) and of a concrete plate reinforced by iron nanoparticles *Vr* = 30% (Figure 9b). The plates are assumed square and simply supported on their line edges. Different loading patterns (single sine, uniformly, concentrated, and linearly distributed loads) are applied on the plates to determine the loading effect on the axial displacement of concrete plates. It is to be noted that the uniform load has a much higher effect on the axial displacement of concrete plates reinforced by iron nanoparticles compared to the other types of loadings, which is the same as the case of transverse displacements mentioned before. Markedly, iron nanoparticles reinforcement in a concrete matrix reduces the non-dimensional axial displacement (*U*) of the composite.

Table 2 shows the non-dimensional normal ($\sigma_{x},\;\sigma_{xy}$) and shear stresses ($\tau_{xz}$) of a simply supported rectangular (*a* = *b*) concrete slab under sinusoidal loading. The concrete slab is reinforced with different volumes of ferric oxide nanoparticles (*V_r_* = 0%, 10%, 20%, 30%), while varying the geometric ratio (*a/h*). The constraints became nearly unchangeable when *a*/*h* exceeded 30.

In Table 2, it can be seen also that the reinforcement concentrations of iron nanoparticles have a very slight effect on the shear stresses ($\tau_{xz}$) which evolve through the thickness of the composite slab. It can be taken that the shear stresses ($\tau_{xz}$) are almost stable when varying reinforcement volumes *(V_r_).* In reverse, the reinforcement concentration (*V_r_*) interestingly decreases the normal stress ($\sigma_{x}$) and increases ($\sigma_{xy}$) of the nano-reinforced plate.

### 4.3. Part II: Thermoelastic Bending

As with mechanical bending, mathematical modeling using the refined trigonometric shear deformation theory (RTSDT) must be verified analytically for predicting the thermoelastic bending of slabs. Since there are no models of concrete slabs reinforced with iron nanoparticles in the literature, it is quite appropriate to check the accuracy of the present mathematical model (RTSDT) using the functionally graded (FG) sandwich-structured plate of Zenkour (2008) [30].

By setting ‘*P* = 0’ and considering the material and geometrical parameters similar to those of Zenkour (2008) [30], the results for the transverse displacements ($\omega_{T}$) as well as the normal and shear stresses ($\bar{\sigma_{x}},\;\bar{\tau_{xZ}}$) of the sandwich plates subjected to a linearly varying thermal load (${\bar{T}}_{2}=100$) through the thickness of the plate are listed for comparison in Table 3.

As observed, the different plate theories are very consistent in predicting thermoelastic bending. Similar displacement, normal, and shear stresses results can be obtained using different shear deformation theories or shape functions.

**Figure 10:**
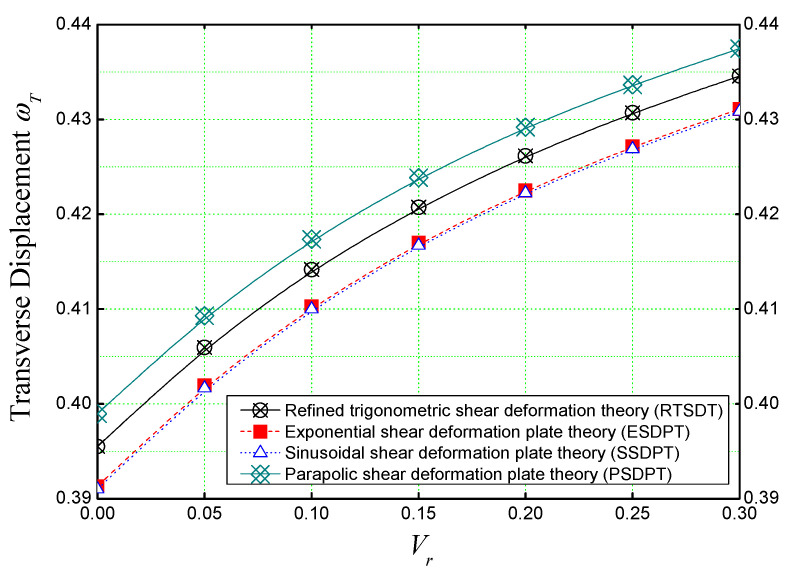
Dimensionless center deflection of Fe_2_O_3_-reinforced concrete slab subjected to linearly varying thermal field (*a/h* = 10, *a* = *b*, ${\bar{T}}_{2}=100$*,* ${\bar{T}}_{3}={\bar{T}}_{1}=0)$.

Figure 10 shows the non-dimensional transverse displacement ($\omega_{T}$) predicted by different plate theories as a function of the concentration of iron nanoparticles *(Vr)* in concrete matrix. The concrete slab is assumed to be simply supported and subjected to a thermal load that varies linearly through the plate thickness (${\bar{T}}_{2}=100,{\bar{T}}_{3}={\bar{T}}_{1}=0)$. The parabolic shear deformation theory (PSDPT) seems to have a slight overestimation over the other plate theories, while the exponential (ESDPT) and sinusoidal (SSDPT) shear theories are corresponding. Otherwise, the plate theories are in accordance when predicting the transverse deflection of the nano-composite concrete slab under thermal load.

In contrast to the case of the mechanical bending, here the reinforcement by nano-iron (Fe_2_O_3_) has a weakening effect on the slab behavior when considering the thermal load. It can be clearly seen that by increasing the volume of nano-iron (*V_r_*), the deflection increases, and this is due to the thermoelastic properties of the nano-iron reinforcements (the high thermal expansion of the iron nanoparticles).

**Figure 11:**
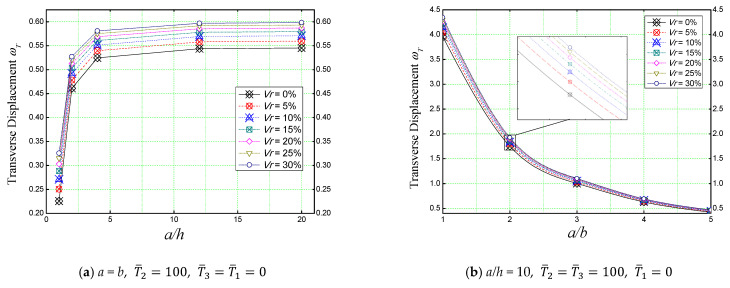
Effect of geometric parameters on the non-dimensional center deflection of a reinforced concrete slab under different thermal loads.

The effect of geometric ratios (*a/h* and *a/b*) on the non-dimensional transverse displacement ($\omega_{T}$) of a simply supported concrete plate reinforced with various proportions of iron oxide (Fe_2_O_3_) nanoparticles is illustrated in Figure 11.

In Figure 11a, the effect of the length/thickness (*a/h*) ratio of a plate subjected to a linearly varying thermal load is shown with regard to the different nanometric ferric oxide incorporation volumes (*V_r_*), whereas Figure 11b shows us the effect of the length/width (*a/b*) ratio of a concrete plate subjected to a combination of linear and non-linear thermal field (${\bar{T}}_{2}={\bar{T}}_{3}=100$).

As mentioned before, these results also confirm nano-iron’s weakening effect on the plate, and this is mainly due to the thermoelastic properties (high thermal expansion) of the nano-iron incorporated in the concrete matrix. Therefore, the higher the percentage of particles present in the concrete matrix, the higher the deflection became, regardless of the geometric ratios.

**Figure 12:**
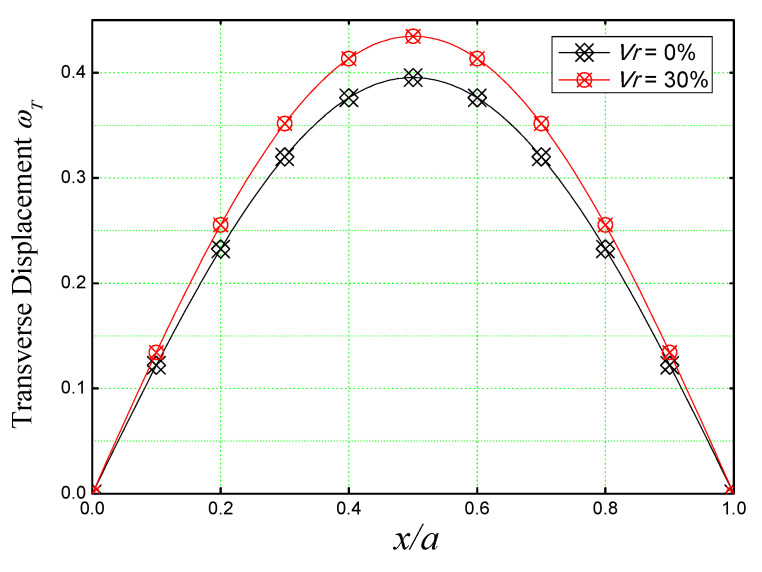
Dimensionless transverse displacement of concrete slab reinforced with Fe_2_O_3_ nanoparticles under linearly varying ${\bar{T}}_{2}=100$ thermal load (*a/h* = 10, *a* = *b*, ${\bar{T}}_{3}={\bar{T}}_{1}=0$).

A comparison between the thermoelastic deflection ($\omega_{T}$) along the length (*x/a*) of an unreinforced concrete slab and a concrete slab incorporated with iron nanoparticles of volume *V_r_* = 30% is shown in Figure 12. Both plates are considered simply supported and subjected to linearly varying thermal load (${\bar{T}}_{2}=100$). It is too clear by seeing Figure 12 that when *Vr* is 30%, the plate deflects even more in comparison to the non-reinforced concrete plate.

**Figure 13:**
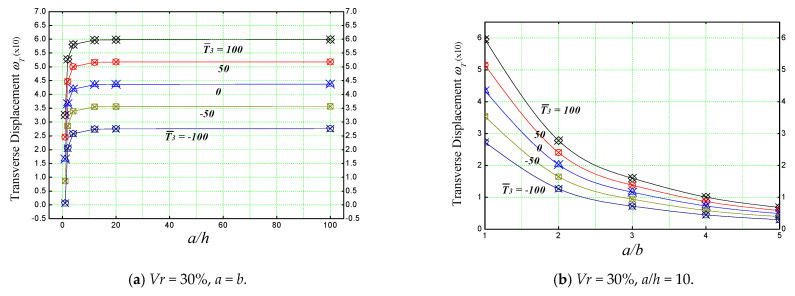
Effect of geometric ratios on the transverse displacement of a concrete slab under different thermal loads.

Figure 13 illustrates the variation of the non-dimensional transverse displacement of a reinforced concrete slab (*V_r_* = 30%) subjected to different thermal loads, as a function of different geometric ratios.

In Figure 13, it can be seen that the length-to-thickness (*a/h*) ratio has an increasing effect on the deflection of the plate. However, when the ratio (*a/h*) exceeds 10, the deflection starts to be stable. On the other hand, the length-to-width ratio (*a/b*) has a sliding effect on the thermoelastic deflection of the plate. It can be noticed also from Figure 13 that the application of combined linear and non-linear thermal loads (${\bar{T}}_{3}=100$) has a greater effect on the behavior of the plate in comparison to the other loads. In general, these results reveal that the variation of transverse displacement is very sensitive to the variation of the thermal load ${\bar{T}}_{3}$ value. 

**Figure 14:**
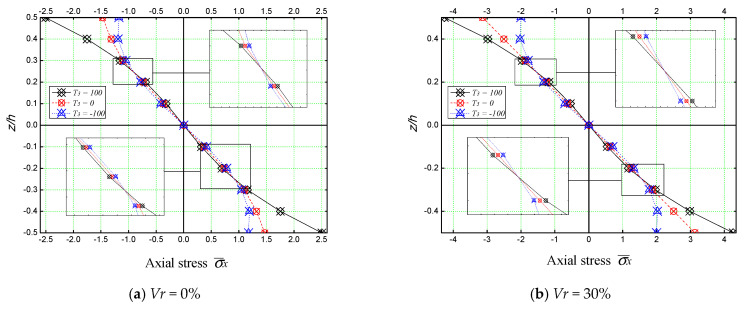
Variations of dimensionless axial stress through the thickness of square plates under different thermal loadings (*a/h* = 5, *a* = *b*).

Figure 14 contains the axial stress ($\bar{\sigma_{x}}$) plot through the thickness (*z/h*) of an unreinforced concrete slab (*V_r_* = 0%) and a ferric oxide Fe_2_O_3_ reinforced concrete slab (*V_r_* = 30%). It is evident that the stresses are tensile below the mid-plane (*z/h* = *0*) and compressive above the mid-plane for both plates (Figure 14a,b). The axial stress is continuous through the thickness of the slab. The results also show a nonlinear variation of the axial stress through the thickness of the plate for both cases. The maximum axial stresses are those generated by the thermal load (${\bar{T}}_{3}=100$).

**Figure 15:**
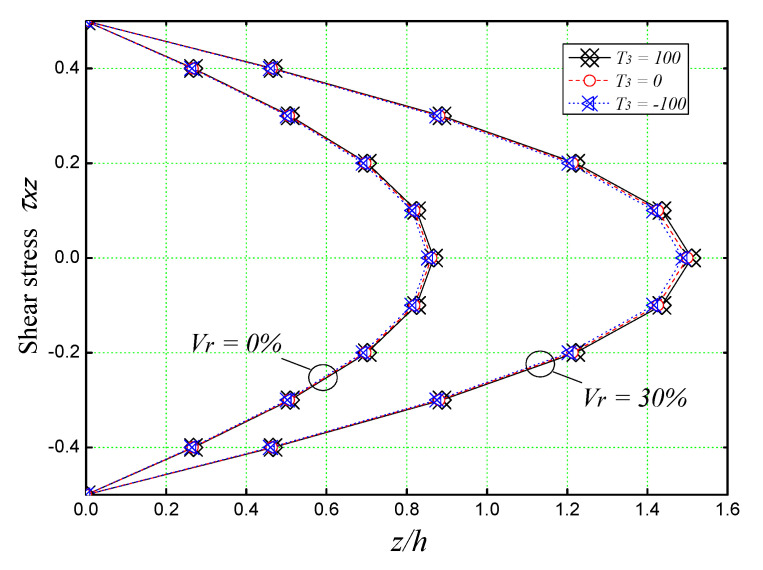
Effect of the thermal loads on the transverse shear stress τ_xz_ of concrete plate reinforced with iron nanoparticles (*a/h* = 5, *a* = *b*).

Figure 15 shows the through-thickness distributions of transverse shear stresses *τ_xz_* in the unreinforced and reinforced concrete plates. It is observed that the maximum value occurs at the center of the plate as in the case of the homogeneous plate. It is also noticeable that the maximum stresses are marked when the reinforcement volume is 30%.

## 5. Conclusions

To design a nano-concrete, it is imperative to perform an analytical model to make a preliminary study on the effect of different added nanostructures on the physical characteristics of concrete.

In this paper, the static behavior of concrete slabs reinforced with iron nanoparticles was analyzed. A refined trigonometric plate theory (RTSDT) was used to simulate the plate, which is assumed simply supported and subjected to different thermal and mechanical loads. The Eshelby homogenization model was used to determine the thermoelastic properties of the nano-composite and virtual work principle to determine the governing equations.

Based on this study, the findings are the following:

- The use of iron nanoparticles as reinforcement in a concrete matrix can improve its performance against external mechanical loads.
- The deflection of iron nanoparticles-reinforced slabs when subjected to different mechanical loads is further decreased by increasing the volume of the nano-iron particles and thickness to length ratios, since the transverse displacement of slabs containing 30% nano-Fe_2_O_3_ was almost 45% lower than that of non-reinforced ones.
- Iron nanoparticles have a greater impact than silica nanoparticles when it comes to strengthening concrete against mechanical loads.
- On the other hand, incorporating iron nanoparticles in a concrete matrix will weaken the nano-composite structure when exposed to thermal loads, where the transverse displacement increases by almost 10%.

Eventually, the authors hope that the outcomes of this investigation will contribute to the mathematical modeling of nanoparticles-reinforced concretes.

## Figures and Tables

**Table 1:** Validation of present theory against other published theories in predicting the mechanical bending of FG plate, (*a/h* = 10, *b* = 3*a*, *P* = 0).

Method	Shape Function *f(z)*	ω	$\sigma_{x}$	$\sigma_{xy}$
Refined Trigonometric Shear Deformation Theory (RTSDT)	$f\left(z\right)=z-\frac{h}{\pi }\sin\left(\frac{\pi z}{h}\right)$	0.2960	1.9955	0.7065
Huu-Tai Thai et al. [40] Refined plate theory.	$f\left(z\right)=-\frac{z}{4}\sin\left(\frac{5z^{3}}{3h^{2}}\right)$	0.2961	1.9943	0.7067
Zenkour [30] Refined Sinusoidal Shear Deformation theory.	$f\left(z\right)=\frac{h}{\pi }\sin\left(\frac{\pi z}{h}\right)$	0.2960	1.9955	0.7066

**Table 2:** Variation of the dimensionless shear stresses $\sigma_{x},\;\sigma_{xy}$, and $\tau_{xz}$ of reinforced concrete square plates using the refined trigonometric shear deformation theory (RTSDT).

***L/h***	*V_r_*= 0%			*V_r_* = 10%			*V_r_* = 20%			*V_r_* = 30%		
	$\sigma_{x}\;$	$\sigma_{xy}\;$	$\tau_{xz}\;$	$\sigma_{x}\;$	$\sigma_{xy}\;$	$\tau_{xz}\;$	$\sigma_{x}\;$	$\sigma_{xy}\;$	$\tau_{xz}\;$	$\sigma_{x}\;$	$\sigma_{xy}\;$	$\tau_{xz}\;$
5	0.1742	1.3934	0.2457	0.1646	1.4832	0.2458	0.1576	1.5496	0.2458	0.1525	1.5981	0.2458
10	0.2462	1.3616	0.2462	0.1611	1.4514	0.2462	0.1543	1.5178	0.2462	0.1494	1.5663	0.2462
15	0.1695	1.3557	0.2463	0.1604	1.4455	0.2463	0.1537	1.5119	0.2463	0.1489	1.5604	0.2463
20	0.1692	1.3536	0.2463	0.1602	1.4435	0.2463	0.1535	1.5098	0.2463	0.1487	1.5583	0.2463
30	0.1690	1.3521	0.2464	0.1600	1.4420	0.2464	0.1534	1.5084	0.2464	0.1485	1.5568	0.2464
60	0.1689	1.3512	0.2464	0.1599	1.4411	0.2464	0.1533	1.5075	0.2464	0.1484	1.5559	0.2464
100	0.1689	1.3511	0.2464	0.1599	1.4409	0.2464	0.1533	1.5073	0.2464	0.1484	1.5557	0.2464

**Table 3:** Validation of present theory against other published theories in predicting the thermo-mechanical bending of FG plate, (*a/h* = 10, *a* = *b*, ${\bar{T}}_{2}=100$ *,* ${\bar{T}}_{3}={\bar{T}}_{1}=0).$

*P*	Shape Function *f(z)*	L/h=10 a=b		
		$\omega_{T}$	$\bar{\sigma_{x}}$	$\bar{\tau_{xZ}}$
1	$f\left(z\right)=z\left(1-\frac{4z^{2}}{3h^{2}}\right)$	0.4803	2.07968	0.57406
	$f\left(z\right)=\frac{h}{\pi }sin\left(\frac{\pi z}{h}\right)$	0.4803	2.28689	0.76244
	$f\left(z\right)=\frac{h}{\pi }sin\left(\frac{\pi z}{h}\right)$	0.4616	2.28689	0.76244
	*f(z)* = z	0.4803	2.07968	/

## Data Availability

The data presented in this study are available on request from the corresponding author.
